# The Transport of Wounded: The Official Descriptive Statement by the Press Bureau

**Published:** 1914-10-03

**Authors:** 


### THE TRANSPORT OF WOUNDED.

### The Official Descriptive Statement by the Press Bureau.

We append below the official statement issued by the Press Bureau, which describes the arrangements made for the conveyance of the wounded from the Front to their various destinations in this country:—

All the hospital-ships proceed to Southampton, where there is a special staff for the reception and distribution of the sick and wounded officers and men who are being sent home on them. The arrangements are under the control of a Surgeon-General, who holds the appointment of a deputy-director of medical services. He has at his command twelve ambulance trains specially constructed for the conveyance of four officers and ninety-six men lying down, or for a considerably greater number of patients sitting up. Twice weekly telegrams are received by him from all the larger military and Territorial Force general hospitals stating the number of beds vacant in each. With this information before him he arranges convoys of sick and wounded on arrival and dispatches them to their destination in one or more of the ambulance trains.

Already the sick and wounded from overseas have been comfortably placed under treatment in most of the large military or Territorial Force hospital centres. At the railway stations of these localities arrangements are made by the military authorities for conveying sick and wounded in motor or other ambulance vehicles from the railway stations to the hospitals. Voluntary Aid Detachments have already done useful work in connection with this stage of the movements of the sick and wounded, and it is expected that the scope for utilising voluntary aid in this direction will be extended as its value becomes better known.

As the military hospitals get filled up arrangements have been made for transferring sick and wounded from them to various hospitals arranged by voluntary effort. Many schemes have been submitted to the War Office, through the British Red Cross Association, in accordance with Field Service Regulations. At present the opportunity of using private hospitals to any great extent has not arisen, as there are still several thousand beds vacant in the military and Territorial Force hospitals. There is no doubt, however, that in time private hospitals will be of much use as an overflow, and also when it is necessary to set free a sufficient number of beds for future requirements in the larger military hospitals.

### The Convalescent.

When sick and wounded are sufficiently convalescent to be granted sick furlough, advantage is being taken of the many offers of accommodation for them in convalescent homes in different parts of the country; and, in order to prevent overlapping and to facilitate the means of placing men on sick furlough, so far as possible, in their own counties, a Central Registry of Convalescent Homes has been formed by a joint committee of the British Red Cross Society and the Soldiers' and Sailors' Help Society. This central registry acts as a clearing-house. Only convalescents who would be given sick furlough to their own homes, if they so desired, are being sent to convalescent homes. Convalescents who require continued hospital treatment will be sent either to the special home in connection with the hospital from which they are transferred (under the supervision of the medical officer of the hospital) or to one or other of the private hospitals already referred to.

In order to enable a convalescent to be placed on sick furlough in a convalescent home, all that he has to do is to inform the medical officer who is in charge of him where and what county or neighhood he would like to proceed to. These particulars are entered on a form and sent to the central registry. The address of the nearest railway station to the convalescent home in the neighbourhood is entered on the form, and it is immediately returned to the medical officer of the hospital. Whenever the convalescent is ready to leave on sick furlough the medical officer sends word to the convalescent home, stating the hour of the man's arrival at the railway station, where arrangements are made to meet and take him over. This arrangement has been working very well, and already over 100 convalescents have been received in various convalescent homes.

It may also be of interest to know that in all the hospitals arrangements are made for replenishing any deficiencies in the men's kits and for giving them any additional clothing which it may be desirable for them to take with them when they go on sick furlough. The hospitals are, for this purpose, receiving many generous gifts of pyjama suits and other articles of clothing.

At the end of their sick furlough the men are required to rejoin the depôts of their regiments in order to be refitted, until arrangements are made for their rejoining their units, either in this country or abroad. They are provided with railway warrants to enable them to go to convalescent homes and to rejoin at their depôts. Arrangements have also been made that they shall receive their pay both while they are in hospital and while they are convalescent.

